# Supplementary material for: Seasonal dynamics of the microbial methane filter in the water column of a eutrophic coastal basin
Source: FEMS Microbiol Ecol. 2024 Jan 27;100(3):fiae007. doi: 10.1093/femsec/fiae007 (PMC10939384; doi:10.1093/femsec/fiae007)
Supplement: fiae007_Supplemental_Files [file fiae007_supplemental_files.zip › Supplementary Information_revision.pdf]

Supplementary Information

Seasonal dynamics of the microbial methane filter in the water column of a eutrophic coastal basin

Jessica Venetz[1], Olga M. Żygadłowska[2], Nicky Dotsios[1], Anna J. Wallenius[1], Niels A.G.M. van Helmond[1,2], Wytze K. Lenstra[1,2], Robin Klomp[1,2], , Caroline P. Slomp[1,2], Mike S.M. Jetten[1] and Annelies J. Veraart[3]

Supplementary methods

The concentrations in the subsamples (c_a) were calculated with Henry's law. Measured headspace concentrations (c_g) were multiplied by Henry solubility coefficient H^{cc} (Sander, 2015):

$$c_a = H^{cc} c_g \qquad [1]$$

With the Henry solubility coefficient is defined as follows: $H^{cc} = H^{cp}RT = \beta \frac{1}{RT^{STP}} RT$ [2]

H^{cp}: Henry solubility coefficient (defined as c_a/p)

R: ideal gas constant (8.314 J mol^{-1} K^{-1})

T: temperature (294.14 K)

T^{STP}: the standard temperature for Bunsen coefficient (273.15 K)

β: Bunsen coefficient (including salinity and temperature)

We accounted for the changes in solubility due to salinity and temperature in the calculation of the Bunsen coefficients (Weiss 1970):

$$ln\beta = A_1 + A_2\left(\frac{100}{T}\right) + A_3 ln\left(\frac{T}{100}\right) + S\left[B_1 + B_2\left(\frac{T}{100}\right) + B_3\left(\frac{T}{100}\right)^2\right] \qquad [3]$$

A_{1-3}, B_{1-3}: Bunsen constants, specific for gas

T: Temperature (294.15 K)

S: Salinity (30 ‰)

The Bunsen coefficient for CH_4, CO_2 and O_2 was calculated according to the specific constants for each gas (Weiss, 1970, 1974; Yamamoto *et al.*, 1976).

Supplementary figures

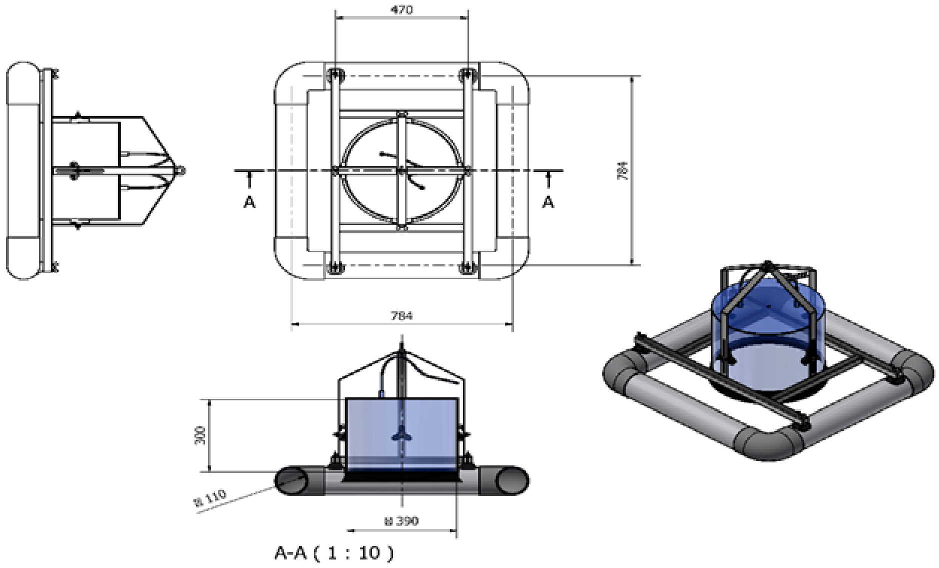

Figure S1: *Construction plan of the floating chamber used for in situ flux measurements. Measurements are given in mm. Construction was planned and made by the TechnoCentrum at Radboud University, Nijmegen (NL).*

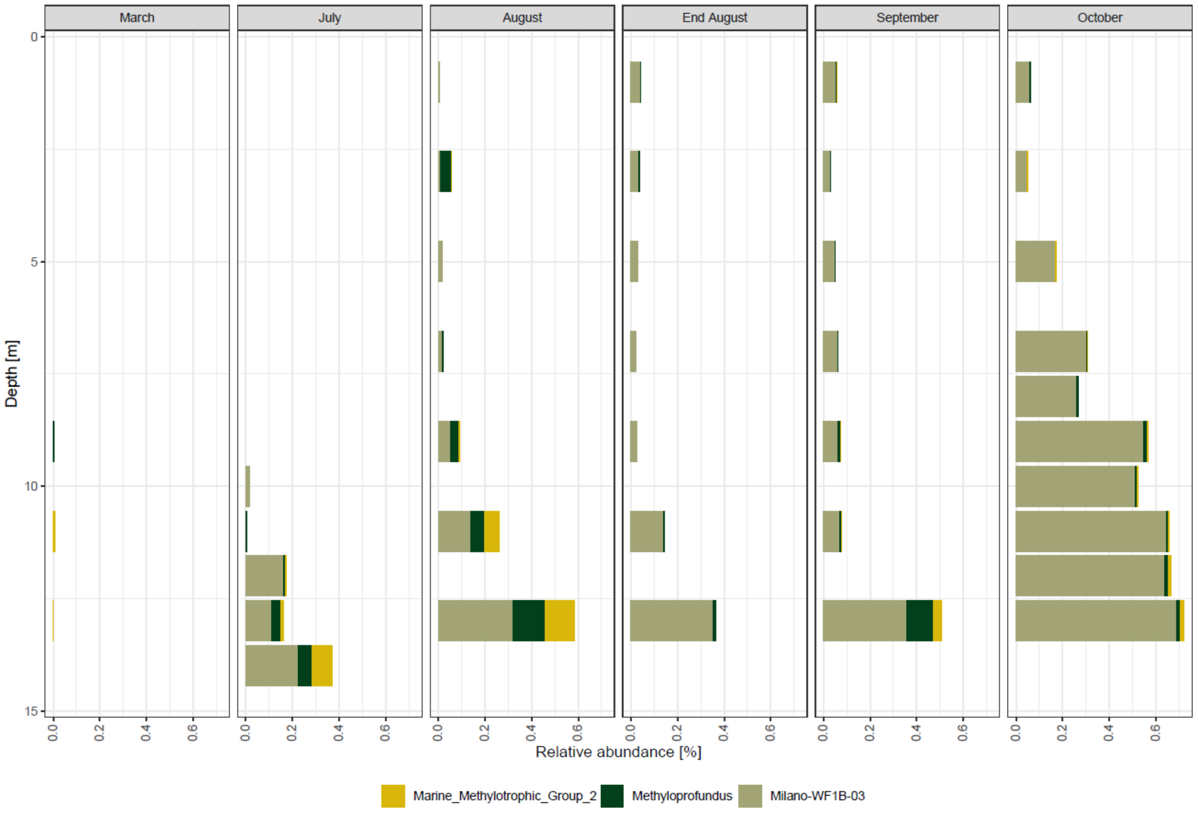

Figure S2: *Depth profiles of relative abundances of MOB genera counts retrieved by 16S rRNA Illumina sequencing.*

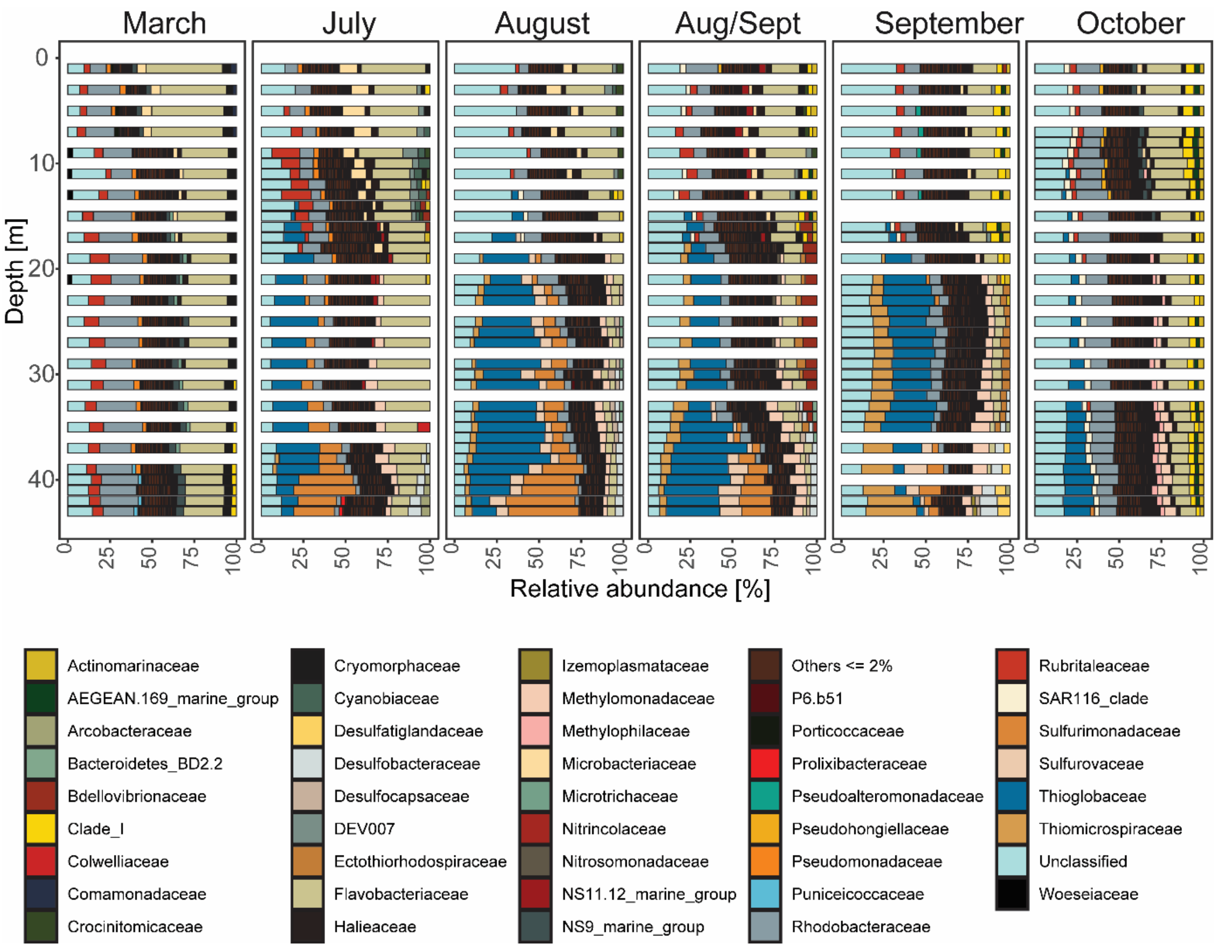

Figure S3: *Depth profiles of relative abundances of bacterial family counts retrieved by 16S rRNA Illumina sequencing.*
